# Rapid Prototyping of Bio-Inspired Dielectric Resonator Antennas for Sub-6 GHz Applications

**DOI:** 10.3390/mi12091046

**Published:** 2021-08-29

**Authors:** Valeria Marrocco, Vito Basile, Ilaria Marasco, Giovanni Niro, Luigi Melchiorre, Antonella D’Orazio, Marco Grande, Irene Fassi

**Affiliations:** 1 STIIMA CNR, Institute of Intelligent Industrial Technologies and Systems for Advanced Manufacturing, National Research Council, Via P. Lembo, 38/F, 70124 Bari, Italy; vito.basile@stiima.cnr.it; 2 Politecnico di Bari, Dipartimento di Ingegneria Elettrica e dell’Informazione, Via E. Orabona 4, 70125 Bari, Italy; ilaria.marasco@poliba.it (I.M.); giovanni.niro@poliba.it (G.N.); l.melchiorre@studenti.poliba.it (L.M.); antonella.dorazio@poliba.it (A.D.); marco.grande@poliba.it (M.G.); 3 Center for Biomolecular Nanotechnolgies, Istituto Italiano di Tecnologia (IIT), Via E. Barsanti 14, 73010 Arnesano, Italy; 4 STIIMA CNR, Institute of Intelligent Industrial Technologies and Systems for Advanced Manufacturing, National Research Council, Via A. Corti, 12, 20133 Milan, Italy; irene.fassi@stiima.cnr.it

**Keywords:** stereolithography, dielectric resonator antennas, Gielis superformula, wideband, sub-6 GHz applications

## Abstract

Bio-inspired Dielectric Resonator Antennas (DRAs) are engaging more and more attention from the scientific community due to their exceptional wideband characteristic, which is especially desirable for the implementation of 5G communications. Nonetheless, since these antennas exhibit peculiar geometries in their micro-features, high dimensional accuracy must be accomplished via the selection of the most suitable fabrication process. In this study, the challenges to the manufacturing process presented by the wideband Spiral shell Dielectric Resonator Antenna (SsDRA), based on the Gielis superformula, are addressed. Three prototypes, made of three different photopolymer resins, were manufactured by bottom-up micro-Stereolithography (SLA). This process allows to cope with SsDRA’s fabrication criticalities, especially concerning the wavy features characterizing the thin spiral surface and the micro-features located in close proximity to the spiral origin. The assembly of the SsDRAs with a ground plane and feed probe was also accurately managed in order to guarantee reliable and repeatable measurements. The scattering parameter S_11_ trends were then measured by means of a Vector Network Analyzer, while the realized gains and 3D radiation diagrams were measured in the anechoic chamber. The experimental results show that all SsDRAs display relevant wideband behavior of 2 GHz at −10 dB in the sub-6 GHz range.

## 1. Introduction

Bio-inspired designs have proved to be effective in the enhancement of antenna performance, especially for microstrip-based ones at higher frequency ranges, such as WLAN, WiMAX, 5G, and mm-wave [1]. A variety of natural shapes and geometries, showing improved bandwidth with reduced sizes and good radiation efficiency, has been widely discussed in research. In particular, flower-shaped patches conceived on a defective ground structure (DGS) [2], Oxalis triangularis plant-leaf shapes for the realization of two frequency-selective surfaces (FSS) [3], Carica Papaya leaf-based patches [4], sunflower-seed-shaped surfaces [5], and fractal and self-similar fractals [6,7,8,9] have demonstrated their potential to achieve large bandwidth, high gain, multiband, and highly directive characteristics. Additionally, a four-leaf clover geometry was exploited to realize patch elements of an antenna array designed for wireless applications [10]. Investment in such nature-inspired shapes has been further promoted by the introduction of Gielis Superformula [11,12], which facilitates the design of patch antennas [13,14,15], metamaterials and metasurfaces [16,17], frequency-selective surfaces (FSS) [18], and split-ring resonators [19,20]. Moreover, besides planar antennas, one of the most recent and intriguing uses of the superformula concerns the design of Dielectric Resonator Antennas (DRAs) [21,22].

Currently, DRAs are proposed as an interesting alternative to planar antennas for 5G and mm-wave applications, due to their ability to satisfy the demand of wide bandwidth and to overcome the issue of the conduction losses experienced by metal-based patch antennas at higher frequencies. In particular, DRAs made of low dielectric constant materials with low losses have proven capable of fulfilling this requirement better than metal-based antennas. Additionally, since polymers can be exploited for DRA fabrication, the opportunity to use Additive Manufacturing (AM) technologies is further increasing the interest in and efforts towards DRA optimization among the scientific community.

As a matter of fact, several dielectric materials are suitable for use in AM technologies, although few of them have actually been evaluated and characterized in the microwave range. Among polymers, the thermoplastic Poly-Lactic Acid (PLA) [23,24,25] and, more recently, photopolymer resins [26], have been analyzed, characterized, and utilized.

Nonetheless, even though low dielectric constant materials such as polymers can ensure DRA wideband behavior, they show drawbacks related to antenna gain and efficiency. For this reason, the use of such materials has implied the exploitation of complex 2.5D and 3D geometries for the design of DRAs.

To this end, some authors proposed a 3D quadrupole-shaped DRA suitable for WLAN (8 GHz) [24] and 5G applications (3.5 GHz) [25], and super-shaped DRAs (S-DRAs), based on the Gielis superformula, operating at 3.5 GHz [26,27,28] and between 6–13 GHz [29], respectively. Basile et al. [26] reported the design and manufacture of a star-based S-DRA and 3D twisted versions of this design operating at 3.5 GHz: the antennas were manufactured by micro-inverted stereolithography (SLA) and realized by means of a photopolymer resin, which was opportunely characterized in the frequency range of interest. The experimental results showed that this kind of S-DRA was able to provide a gain around 3 dBi and a radiation efficiency of ~90% at 3.5 GHz, with a significant reduction of the antenna volume and weight. Simeoni et al. [28] reported a classic 2.5D star-shaped S-DRA antenna made of polyvinyl chloride (PVC)—a thermoplastic material with low permittivity (ε_r_ ≈ 2.8) and negligible tangent loss—fabricated by means of the extrusion process. The antenna exhibited a fractional bandwidth of 74% and a gain of ~8 dBi between 6 and 13 GHz.

Recently, spiral-based geometries were also considered for DRAs. Indeed, spiral and Fibonacci-based shapes have been frequently exploited in the antenna scenario to tailor planar and metal-based antenna performance. Fibonacci spirals were used for FSS implementation in microwave absorbers [30] and radome applications [31], or applied to modified Koch curves for antenna miniaturization [32]. Furthermore, Logarithmic spiral slots have been used for the implementation of cylindrical DRA multimode operations [33]. In addition, the design of a Spiral shell DRA (SsDRA), resembling the shell of a sea mollusk, has been presented [34]: the numerical results showed that the optimized antenna was capable of wideband behavior (up to more than 2 GHz, between 3 GHz and 6 GHz), with satisfactory gain and efficiency. Recently, an example of a one-round spiral shell realization was proposed [35]. Due to its small size (height of 12.5 mm and thickness of 3.5 mm), the spiral shell was manufactured by means of SLA. Finally, this element was filled with water and then mounted on a cylindrical DRA to generate multimode orbital angular momentum (OAM) waves at frequencies between 4.8 and 6.36 GHz.

However, besides the aforementioned studies, the fabrication of full spiral-shape-based DRAs is still unaddressed by research, which is reasonable because of the complexity of their manufacture.

In the present paper, the fabrication and the characterization of the optimized SsDRA design presented in by Melchiorre et al. [34] are reported: in particular, three prototypes were manufactured by exploiting different photopolymer resins. In Section 2, the modeling of the antenna geometry is shown. The challenges and criticalities exhibited by the SsDRA’s fabrication are described and discussed in Section 2.1, along with the details of the prototype modeling and manufacture, carried out by means of bottom-up micro-SLA. The assembly of the SsDRA was performed to ensure reliable and repeatable measurements: in this viewpoint, the required positioning accuracy, the mechanical fixation on the ground plane, and the correct feed probe positioning were carefully considered and implemented. Furthermore, the smallest micro-features of the antenna were characterized via a visual system setup to estimate the achieved geometric accuracy. The experimental measurements of the scattering parameter S_11,_ realized gain and 3D radiation diagrams are reported and commented on in Section 3. Finally, additional numerical analyses are reported in Appendix A in order to compare the antenna performance and structural stiffness of the proposed SsDRA in comparison with the common Logarithmic spiral shell DRA (LsDRA).

## 2. The Ggeometrical Modeling, Fabrication, and Geometrical Characterization of the SsDRA Prototypes

The SsDRA was modeled starting from the generation of the curves on the plane, obtained by means of the Equation (1) in the range *θ* ∊ [−3π, +3π]:

(1) $$\mathrm{GT}=\rho \left(\theta ,\;f\left(\theta \right),\;a,\;b,\;m_{1},\;m_{2},\;n_{1},\;n_{2},\;n_{3}\right)=K\cdot f\left(\theta \right)\cdot {\left[{\left|\frac{1}{a}\cos\left(\frac{m_{1}}{4}\theta \right)\right|}^{n_{2}}+{\left|\frac{1}{b}\sin\left(\frac{m_{2}}{4}\theta \right)\right|}^{n_{3}}\right]}^{-{\frac{1}{n}}_{1}}$$

The values of the Gielis superformula set for the optimized SsDRA design are reported in Table 1. The *a* and *b* parameters influence the size and volume of the DRA and, as a consequence, they have an impact on the footprint that it can occupy. Additionally, the increase of such values leads to the proportional increase of the shell wave amplitudes on each loop, while, by setting *a* = *b*, the angular position of the shell wave remains constant. By taking into account all these considerations, the values of *a* = *b* = 1 allow to keep the DRA as compact as possible within the ground plane (100 mm × 100 mm) [34]. The shell wave number over the base spiral depends proportionally on m_1_ and m_2_ values, while *n*_1_, *n*_2_, and *n*_3_ parameters have an effect on the shell wave amplitude on each loop. It is worth noting that by fixing *n*_1_ = *n*_2_ = *n*_3_ = 2, the resulting curve corresponds to the well-known Archimedean spiral. Conversely, when the values of *n*_1_, *n*_2_, and n_3_ are higher than 2, the basic spiral is modulated by alternate waves featuring concavity and convexity, introducing the bio-inspired “nature”. The amplitude and the modulation of the overall wavy spiral are determined by the function f(*θ*) [34,36]:

f(*θ*) = *e*
^(^*^c^_theta_^θ^*^)^ (2)

**Figure 1:**
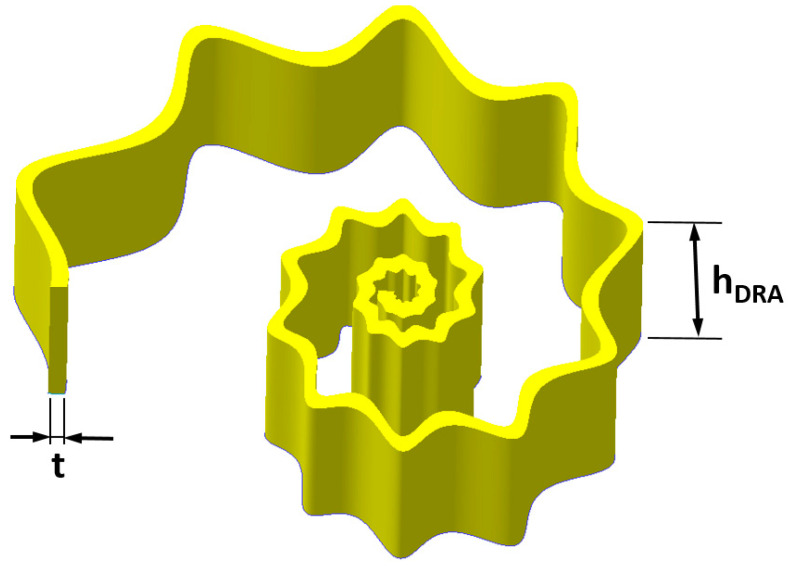
**A** 3D model of the Spiral shell Dielectric Resonator Antenna (SsDRA).

In particular, the geometrical parameters that drive the evolution of the spiral are *K* and *c_theta_*: the higher *K* and *c_theta_*, the higher the amplitude of the spiral radius *ρ*. All values reported in Table 1 were chosen in order to obtain a wider DRA bandwidth and a volume as compact as possible; furthermore, the thickness and width of the DRA were also chosen by considering the antenna stiffness and its fabrication feasibility. In order to provide the shell thickness, *t*, an offset between the internal and external curves was applied. The 3D solid model of the SsDRA, generated by the CAD software Solidworks 2017, was obtained by the extrusion of the closed curves considering a quantity equal to *h_DRA_* (Figure 1).

### 2.1. The Fabrication, Geometric Characterization, and Assembly of the SsDRA Prototypes

The proposed optimized SsDRA is characterized by the following main overall dimensions: thickness *t* = 2 mm, DRA height *h_DRA_* = 29 mm, and a feed probe (pin) height of *h_PIN_* = 17 mm. According to the numerical results reported by Melchiorre et al. [34], it should be considered that the SsDRA’s thickness, *t*, and height, *h_DRA_*, values have a great influence on the trend of the scattering parameter S_11_, especially when it comes to accomplishing 2 GHz bandwidth between 3 and 6 GHz. Indeed, it was observed that, for *t* < 2 mm and *h_DRA_* < 29 mm, the SsDRA experienced bandwidth shrinkage, along with reduced gain. Therefore, the geometrical resolution required for these specific dimensions was 0.5 mm.

**Figure 2:**
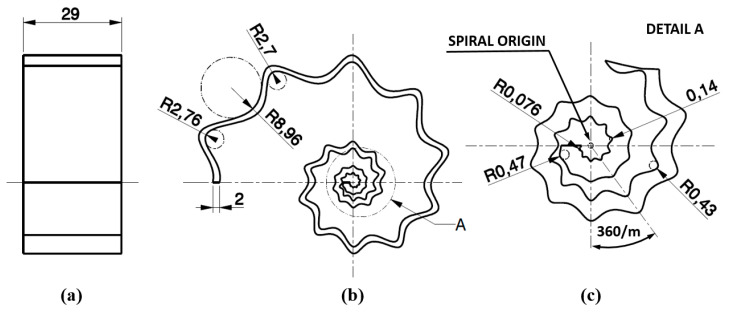
SsDRA Drawings: (**a**) Side view; (**b**) Top view; (**c**) Detailed view of the SsDRA close to the antenna origin. Units: mm and degree.

However, as shown in Figure 2, the waves superimposed on the logarithmic spiral curve introduced an additional criticality in the manufacture of these antenna, as the realization of the wavy spiral loops in proximity to the SsDRA origin plays a key role in the antenna’s performance. Indeed, as can be inferred from the electric and magnetic field distribution at 3.5 GHz reported by Melchiorre et al. [34], the highest field intensities are gathered around the pin location and comprised within the first two loops. In the present case, as *m* = *m*_1_ = *m*_2_ = 10, the ridges and valleys near the spiral origin grew very small and close to each other (Figure 2b,c): indeed, the minimum internal and external estimated radii were 76 µm and 470 µm, respectively. This suggests that the manufacturing process is capable of providing the minimum resolution dictated by the smallest estimated radius. Typically, Digital Light Processing (DLP), Stereolithography (SLA), and micro-droplets deposition technology, such as Polymer Jetting Modeling (PJM), are capable of these low resolutions [37].

In this study, bottom-up micro-Stereolithography (SLA), implemented by the 3D printer Formlabs Form3, was exploited for the SsDRA’s prototyping [38]. For their realization, three photopolymer resins were used: Clear V04 resin (DRA1), Grey V04 resin (DRA2), and Tough blue V05 resin (DRA3) [39].

**Figure 3:**
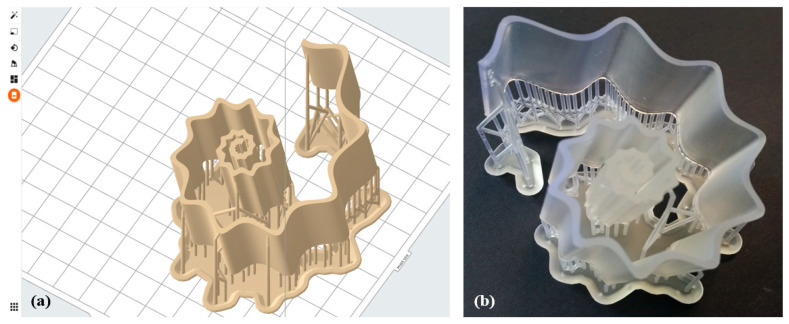
SsDRA fabrication: (**a**) The final model of the SsDRA generated in the Formlabs Preform V3.12.2 software environment; (**b**) The 3D-printed SsDRA prototype made of clear resin (DRA1) before the post-processing phases.

The geometrical solid model of the antenna was exported in Standard Triangulation Language (STL) format by choosing the highest quality export parameters: chord height 0.0098 mm and angle control 1.0. The STL format was then imported into the Formlabs Preform v. 3.17.0 slicing software, in order to generate the file for the manufacturing process (Figure 3a).

In order to guarantee the high quality of the prototype’s printability and to reduce the peeling force applied to each layer during the printing phase, the model was also slanted 15 deg. As can be inferred from Figure 3, this operation was accomplished by foreseeing the presence of additional supports, generated with a medium density index of 1.0 (min 0.5; max 1.5), and featuring attachment points with diameters of 0.7 mm.

The photo-polymerization process of the liquid mixtures was activated by a class 1 violet laser operating at a wavelength of 405 nm and with a power setting power equal to 250 mW. The printing of the final prototype, depicted in Figure 3b, was performed by means of a layer-by-layer strategy implemented along the *z*-axis (height of the structure): the set layer thickness was 100 µm, and the final structure comprised 541 layers. Finally, the SsDRA prototype underwent several post-processing operations: supports removal, washing in high-purity (99.9%) Isopropyl Alcohol (IPA), and additional UV curing (wavelength *λ* = 405 nm, power 39 W) with the parameters reported in Table 2.

Each SsDRA 3D-printing processing time was about 5 h, while the post-processing time (washing and UV curing) took up to 1.5 h.

**Figure 4:**
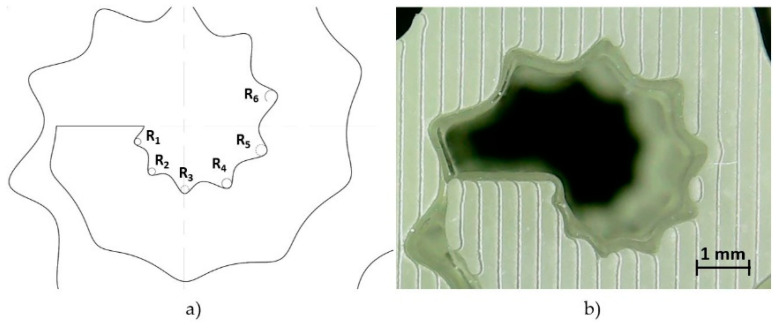
Geometric characterization of the SSDRA: (**a**) evidence of the radii (from R1 to R6) approximating the hollows of the internal SsDRA curve; (**b**) image acquisition of the same micro-features close to the SsDRA’s origin.

In order to evaluate the accuracy of the micro-features close to the SsDRA’s origin, the measurements of the radii approximating the hollow waves were performed by means of a vision system [40]: the images were acquired via a digital microscope (Texon U500×, Texon, Guangzhou, China) with 500× maximum magnification and a resolution of 640 × 680 pixels (0.3 Mpixels). The radii from R1 to R6, highlighted in Figure 4a, were calculated by measuring circumferences of the best-fitting circles on the SsDRA’s edge. In order to frame the inner radii better, the Field of View (FoV) was set equal to 7.32 × 4.49 mm^2^, while the measurement scale was fixed at 87.377 pixels/mm. This vision system setup made it possible to guarantee a spatial resolution, R_s_, equal to 11.44 µm and a feature resolution, R_f_, of 34.32 µm.

The radii measurements were carried out by image processing using ImageJ v.1.53e software, developed by the National Institute of Health NIH, 9000 Rockville Pike, Bethesda, Maryland 20892, USA [41]. The raw image shown in Figure 4b was first subjected to an edge detection algorithm, implemented by a Hue Saturation and Brightness (HSB) filter, set to (255,255,171) of the default Modified Isodata method. The estimates were then compared to the nominal values. The results are summarized in Table 3: it is notable that the final geometric accuracy ranges between −5 µm and +10 µm, which can be considered satisfactory from the process viewpoint. Nonetheless, it is worth stressing that these values are also affected by the intrinsic error pertaining the visual system setup.

**Figure 5:**
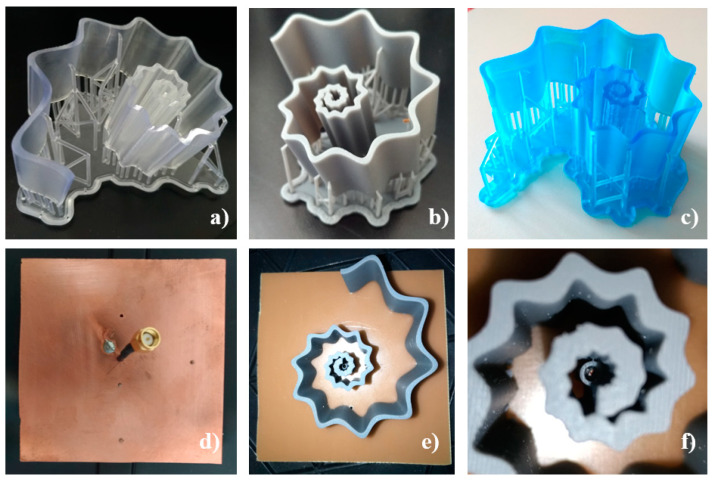
SsDRA prototypes: (**a**) Clear V04 resin SsDRA (DRA1); (**b**) Grey V04 resin SsDRA (DRA2); (**c**) Tough blue V05 resin SsDRA (DRA3); (**d**) Coaxial cable, SMA connector, and pins on the ground plane; (**e**) Top view of the assembled SsDRA prototype; (**f**) Detailed view of the SsDRA’s origin with feed guides.

The spiral geometry of the SsDRA introduced an additional issue regarding the assembly of the ground plane and the positioning of the feed probe. Small rotations of the SsDRA structure could result in considerable displacements of the spiral walls, deviations of the SsDRA position from the nominal one, as well as inaccuracy in feed positioning, thus inducing relevant inaccuracy in the electromagnetic characterization. Therefore, in order to avoid this drawback, four fixed-point constraints were introduced in the assembly: small cylindrical pins (diameter 1.5 mm; height 3 mm) were placed at the base of the SsDRA, and inserted into holes drilled on the ground plane. This solution (Figure 5d) also made it possible to achieve the required mechanical fastening of the SsDRA to the ground plane. Furthermore, in order to obtain an accurate and mechanically stable positioning of the feed probe during measurements, two cylindrical guides (diameter 1.5 mm), placed at different heights, were introduced into the SsDRAs origin (Figure 5e,f).

The 3D-printed SsDRAs representing the Clear V04 resin (DRA1), Grey V04 resin (DRA2), and Tough blue V05 resin (DRA3) prototypes are depicted in Figure 5a–c. The final antennas were assembled at the center of a 1.4 mm thick FR4 substrate, comprising dimensions of 100 mm × 100 mm (Figure 5d–f). A 1.5 mm diameter hole was drilled in the center of the substrate, in correspondence with the antenna origin: here, a coaxial cable was inserted at a height of 17 mm (h_PIN_) to feed the SsDRA. The SMA connector shield was soldered on the copper-coated backside of the substrate (Figure 5d).

## 3. Measurements of the Scattering Parameter S_11_, Realized Gain, and 3D Radiation Patterns

**Figure 6:**
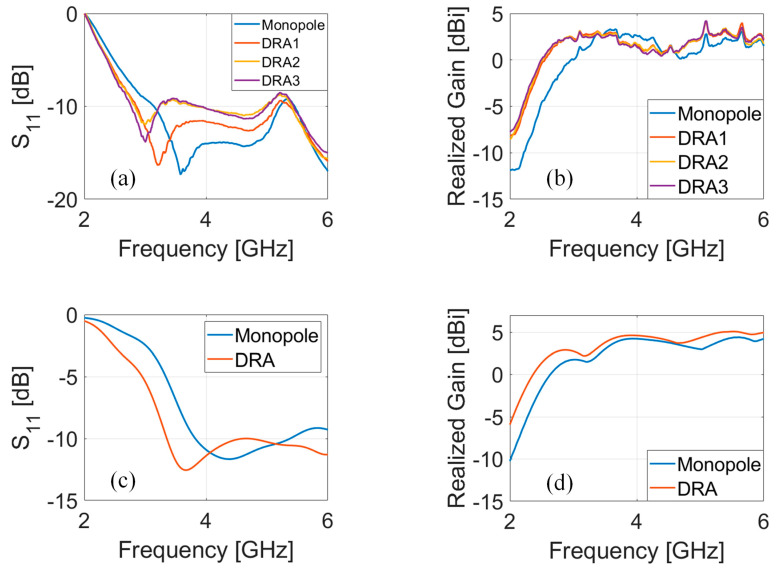
(**a**) Measured scattering parameter S_11_ and (**b**) measured realized gain concerning monopole (blue curve), Clear V04 resin (DRA1, red curve), Grey V04 resin (DRA2, yellow curve), and Tough Blue V05 resin (DRA3, purple curve); (**c**) simulated scattering parameter S_11_ and (**d**) simulated realized gain concerning monopole (blue curve) and Grey V04 resin (DRA, red curve).

The DRA prototypes were first characterized in terms of the scattering parameter S_11_, which was measured by means of a Vector Network Analyser (VNA, Keysight N9917A, Keysight Technologies, 1400 Fountaingrove Parkway, Santa Rosa, CA, USA). Figure 6a,b show the experimental results, while Figure 6c,d report the simulated results pertaining to the SsDRA and the monopole. Figure 6a depicts the scattering parameter S_11_ pertaining to all SsDRAs, e.g., DRA1, made of Clear resin (red curve), DRA2, made of Grey resin (yellow curve), and DRA3, made of Tough Blue resin (purple curve), along with the monopole (blue curve), reported for reference. As can be observed, the dips of the DRAs shifted slightly: DRA2 and DRA3 displayed a resonance of around 3 GHz, while DRA1 experienced a resonance of 3.3 GHz. This slight difference can be imputed to the permittivity value of the three resins: in particular, as Grey and Tough Blue resins are characterized by ε_r_ = 2.7 and tanδ = 0.003 [26], it is reasonable to presume that Clear resin should have slightly lower ε_r_, likely due to a lack of ceramic pigmentation. As can be seen, all antennas exhibited significant wideband behavior of about 2 GHz at −10 dB, corresponding to a fractional bandwidth of ≈ 50%. The realized gain of each SsDRA was measured in the anechoic chamber (StarLab from Satimo, MVG Italy, local supplier: via Castelli Romani, 59, 00071 Pomezia, RM, IT) and reported in Figure 6b: as can be inferred from the curves, all SsDRAs showed gain values equal to 3.5 and 4 dBi, at 3.3 and 5.3 GHz, respectively, providing an increase of +1 dB compared to the monopole. At the same time, the introduction of the SsDRAs made it possible to achieve a positive gain from 2.5 GHz up to 6 GHz. Figure 6c,d show the scattering parameter S_11_ and the realized gain of the analyzed DRA (red curve) and the monopole (blue curve). The numerical analyses were performed by considering the permittivity of Grey resin V04 and neglecting the material losses. As can be seen from the curves, the experimental and numerical analyses were in good agreement. It was also verified that the dimensions (external and internal diameters) of the simulated coaxial cable had a significant impact on S_11_’s behavior, thereby also inducing a slight shift in the SsDRA and monopole dips, while they had a negligible effect on the realized gain.

**Figure 7:**
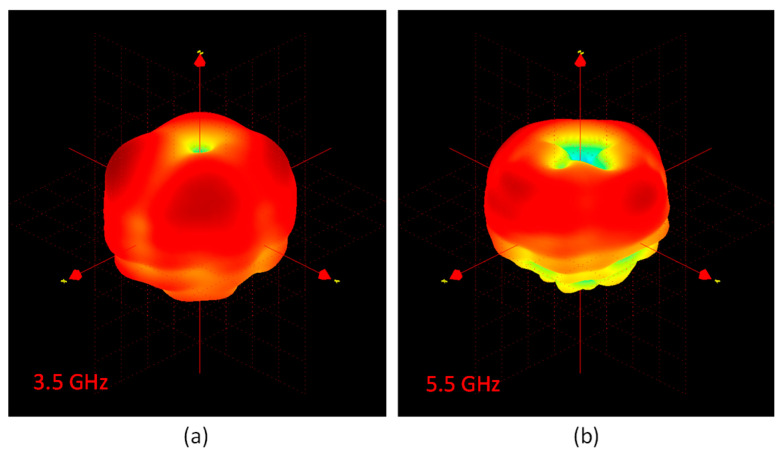

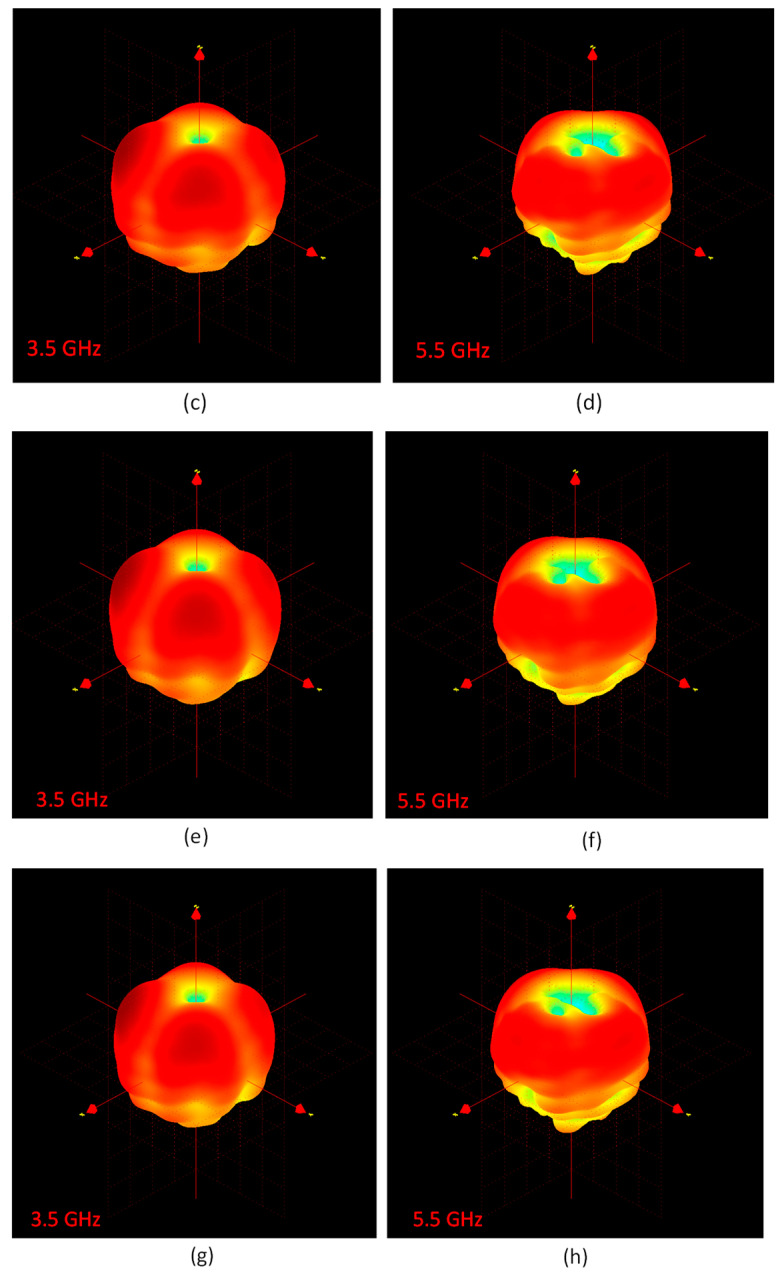
3D radiation patterns: monopole at (**a**) 3.5 GHz and (**b**) 5.5 GHz; DRA1 at (**c**) 3.5 GHz and (**d**) 5.5 GHz; DRA2 at (**e**) 3.5 GHz and (**f**) 5.5 GHz; DRA3 at (**g**) 3.5 GHz and (**h**) 5.5 GHz.

The 3D radiation patterns related to the monopole, DRA1, DRA2, and DRA3, were also measured in the anechoic chamber at two different frequencies, 3.5 and 5.5 GHz, and reported in Figure 7. The plots reveal that all DRAs displayed monopole-like behavior, as expected. Nonetheless, a slight asymmetry in the main lobes was detected for the DRAs: this trend is in line with the simulated results that Melchiorre et al. reported [34].

Finally, in order to highlight the benefits of exploiting bio-inspired geometries on antenna performance, the comparison between the experimental results concerning some examples of planar antennas and DRAs having various shapes is reported in Table 4: in particular, circular and modified bio-inspired patch antennas [1], regular rectangular patches made with different metals [3], classic rectangular and cylindrical DRAs [26], star-shaped DRA [26], and the proposed SsDRA, made with the same photopolymer resin, were considered. All data refer to the frequency range of interest, 2–6 GHz.

As can be inferred from the summarized results, the circular monopole patch had a higher bandwidth than the jasmine flower patch, but at the cost of its larger size (it was 11.30% larger than the latter) [1]. The two rectangular patches made of copper and silver had a very small bandwidth compared to the other antennas. In relation to DRAs, bio-inspired geometry demonstrated its capability of improving the bandwidth in a relevant fashion. Moreover, for the sake of completeness, we highlight that, as was also evidenced by da Silva Júnior et al. [1], in planar antennas, the use of bio-inspired geometry for DRAs leads to a reduction of the antenna volume compared to classic shapes [26,34].

## 4. Conclusions

In this study, the fabrication process and characterization of bio-inspired SsDRAs, whose design is based on the Gielis superformula, were presented. The selection of the most suitable manufacturing process was performed by assessing the required resolution dictated by the wavy spiral arms of the DRA’s geometry. In particular, as the radius values of the spirals diminished from 9 mm down to 76 µm, the dimensional resolution was driven by the smallest radius characterizing the circle in the proximity of the antenna origin. For this reason, bottom-up micro-stereolithography (SLA) was selected to fabricate three SsDRA prototypes, made of three different photopolymer resins. The geometric characterization of the SsDRA prototypes was performed via a visual system setup, showing that the accuracy of the radii, characterizing the smallest micro-features close to the antenna origin, ranges between −5 µm and +10 µm. The prototypes were also characterized by an accurate assembly procedure, with the aim of allowing reliable and repeatable measurements. The antennas were then assembled on a FR4 substrate and fed by an SMA inserted in correspondence with the antenna feed site. The scattering parameter S_11_ highlighted that all SsDRAs have a significant wideband behavior of 2 GHz at -10 dB, considering a central frequency of 4 GHz, while the realized gain values were 3.5 dBi at 3.3 GHz and 4 dBi at 5.3 GHz. The 3D radiation patterns measured at 3.5 and 5.5 GHz show that all antennas displayed monopole-like behavior.

## Figures and Tables

**Table 1:** The Gielis parameters used in Equation (1) to generate the SsDRA’s geometry.

f(*θ*)	*K*	*c_theta_*	*a*	*b*	*m*_1_	*m*_2_	*n*_1_	*n*_2_	*n*_3_
$e^{C_{theta}\cdot \theta }$	6	0.2	1	1	10	10	5	5	5

**Table 2:** UV curing post-processing parameters.

SsDRA	Material	Color	Time (min)	Temperature (°C)
DRA1	Clear V04	Transparent	15	60
DRA2	Grey V04	Grey	30	60
DRA3	Tough V05	Blue	60	60

**Table 3:** Estimate of the radii close to the SsDRA origin: nominal values, measured values, and deviations from nominal values.

Dimension	Nominal Value	Measured Value		Deviation	
	[µm]	[pixels]	[µm]	[µm]	[%]
R1	76	7.252	83.0	7.0	9%
R2	101	8.752	100.2	−0.8	−1%
R3	94	7.752	88.7	−5.3	−6%
R4	104	9.500	108.7	4.7	5%
R5	121	11.500	131.6	10.6	9%
R6	131	12.005	137.4	6.4	5%

**Table 4:** Performance comparison of planar antennas and DRAs with various geometries: circular and modified bio-inspired patch antenna, regular rectangular patches with different metals, rectangular DRA (RDRA), cylindrical DRA (CDRA), star-shaped DRA, and bio-inspired SsDRA.

Ultra Wideband Antennas	Resonant Frequency (GHz)	Bandwidth (GHz) (Up to 6 GHz)	Gain (dB) (Up to 6 GHz)
Circular monopole patch [1]	2.81	2.9	NA
Jasmine Flower patch [1]	3.75	2.25	NA
Rectangular Cu Patch [3]	2.50, 3.85	0.07@2.5, 0.105@3.85	NA
Rectangular Silver Patch [3]	2.42, 3.77	0.11@2.42, 0.12@3.77	NA
C-DRA [26]	3.5	1.28	4.4
R-DRA [26]	3.5	1.16	4.4
S-DRA [26]	3.3	1.32	4.3
Bio-inspired SsDRA	3.3, 5.3	2	3.5, 4.0

**Figure A1:**
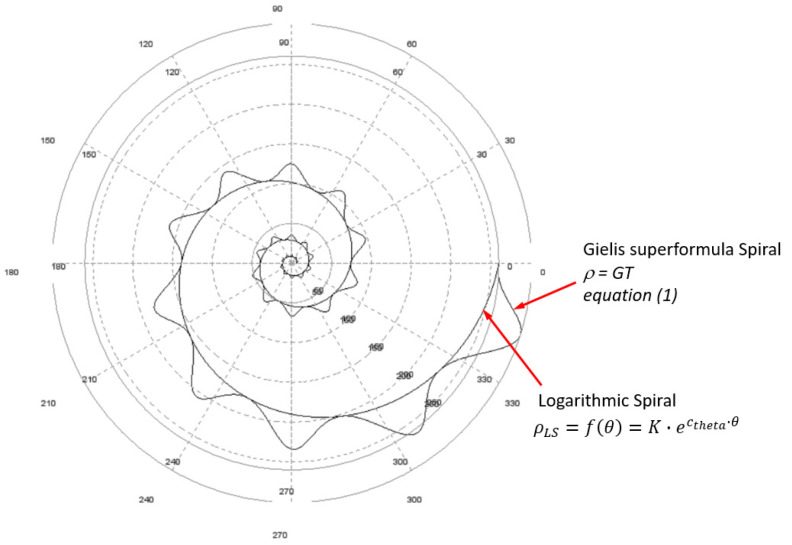
Overlapping of Logarithmic and Gielis superformula spirals with parameters reported in Table 1.

**Figure A2:**
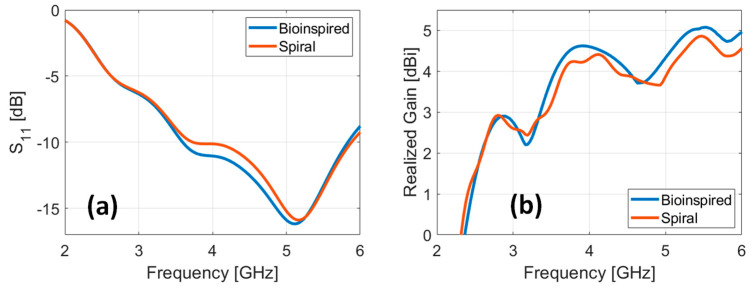
(**a**) Scattering parameter S_11_ and (**b**) gain of the bio-inspired SsDRA (blue curve) and LsDRA (red curve).

**Figure A3:**
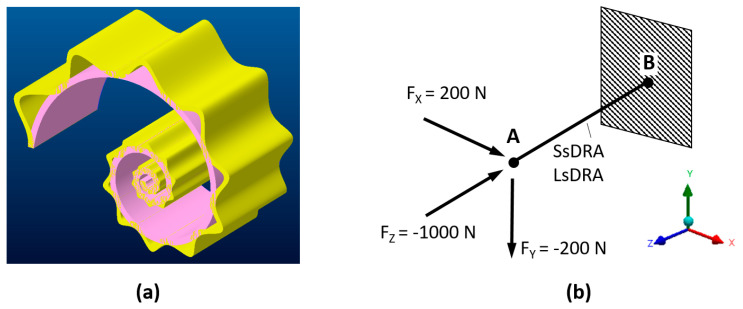
(**a**) Overlapping of equivalent SsDRA and LsDRA models; (**b**) cantilever configuration with a fixed support in B and loads applied in A used for FEM analysis.

**Figure A4:**
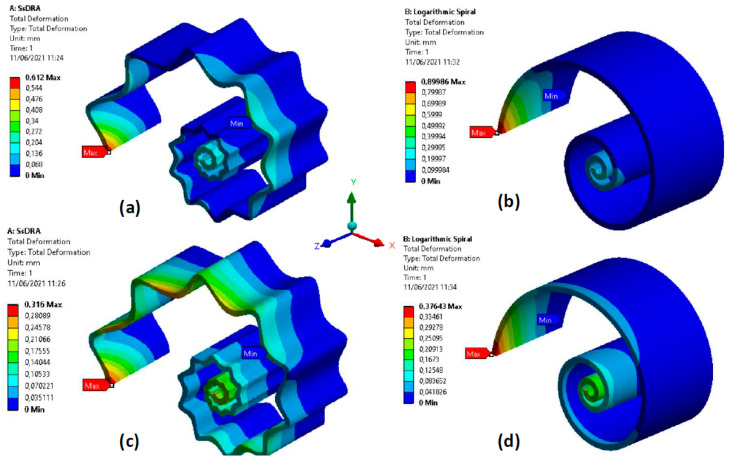
A comparison of the flexural stiffnesses of the SsDRA and LsDRA in cantilever configuration: (**a**) and (**b**) deformations under a load corresponding to Fx = 200 N; Fy = Fz = 0; (**c**) and (**d**) deformations under a load corresponding to Fy = −200 N; Fx = Fz = 0.

**Figure A5:**
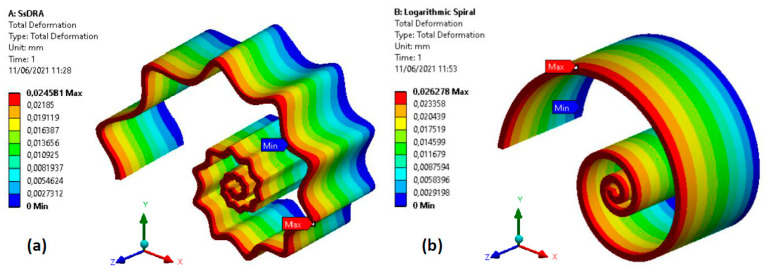
A comparison of the compression stiffness (upsetting) of the (**a**) SsDRA and (**b**) LsDRA: deformations under a load corresponding to Fz = −1000 N; Fx = Fy = 0.

## Appendix A. Supplementary Discussion: SsDRA vs. Logarithmic Spiral Shell DRA (LsDRA)—Antenna Performance and Structural Comparison

In order to show the advantages provided by the SsDRA design, we compared the proposed SsDRA to a Logarithmic spiral shell DRA (LsDRA) (Figure A1): for the simulations, the same overall dimensions and grey photopolymer resin material were considered for both antenna geometries.

The antenna’s performance was evaluated in terms of scattering parameter S_11_ and gain. The numerical results, reported in Figure A2, show that the SsDRA has a slightly higher bandwidth (+0.5 GHz at −10 dB around 4 GHz) compared to the LsDRA, while the SsDRA gain increases by about +0.5 dB at 4 and 5.5 GHz compared with the LsDRA gain.

From a structural perspective, the SsDRA displayed better performance in terms of flexural and compression stiffness. In order to prove the structural improvements brought by the SsDRA with respect to the LsDRA, static structural Finite Element Method (FEM) analyses in a cantilever configuration (Figure A3) were performed by means of ANSYS Workbench 2019 R1 software; the photopolymer resin was modeled according to the material datasheet [36] and the applied postprocessing. The mechanical analysis was carried out by applying loads below the yield limit, e.g., in the linear elastic range. Figure A4 and Figure A5 show the deformations that both DRAs experienced when different force values were applied along each direction; all results are summarized in Table A1. The SsDRA displayed more flexural stiffness along x and y directions (+47% and +19%) compared to the LsDRA; furthermore, the evaluation of the compression stiffness (application of the force along the *z*-axis) showed a better performance by the SsDRA (+8%).

**Table A1:** Summary of the FEM structural analysis of the SsDRA and LsDRA and related stiffnesses.

Load Type	Configuration	Load	Max Displacement δ_MAX_/Stiffnesses k				Variations	
			SsDRA (1) Equation (1) Table 1		LsDRA (2) Equation (1) Table 1 (*n_i_* = 1; *m_i_* = 0)		*Δ_12_* %	
			*δ _MAX_* (mm)	*k* (N/mm)	*δ _MAX_* (mm)	*k* (N/mm)	*Δδ_MAX_*%	*Δk*%
Flexural along x	Cantilever	F_X_ = 200N; F_Y_ = F_Z_ = 0	0.612	326.80	0.900	222.22	−32%	+47%
Flexural along y	Cantilever	F_Y_ = −200N; F_X_ = F_Z_ = 0	0.316	632.91	0.376	531.91	−16%	+19%
Compression	Cantilever	F_Z_ = −1000N; F_X_ = F_Y_ = 0	0.024	41666.67	0.026	38461.54	−8%	+8%
